# Transperineal prostate brachytherapy, using I-125 seed with or without adjuvant androgen deprivation, in patients with intermediate-risk prostate cancer: study protocol for a phase III, multicenter, randomized, controlled trial

**DOI:** 10.1186/1471-2407-10-572

**Published:** 2010-10-21

**Authors:** Kenta Miki, Takayoshi Kiba, Hiroshi Sasaki, Masahito Kido, Manabu Aoki, Hiroyuki Takahashi, Keiko Miyakoda, Takushi Dokiya, Hidetoshi Yamanaka, Masanori Fukushima, Shin Egawa

**Affiliations:** 1 Department of Urology, Jikei University School of Medicine, Tokyo, Japan; 2 Translational Research Informatics Center, Kobe, Japan; 3 Department of Radiology, Jikei University School of Medicine, Tokyo, Japan; 4 Department of Pathology, Jikei University School of Medicine, Tokyo, Japan; 5 Department of Radiation Oncology, Saitama Medical University, Hidaka, Japan; 6 Institutes of Preventive Medicine, Kurosawa Hospital, Takasaki, Japan

## Abstract

**Background:**

The optimal protocol for ^125^I-transperineal prostatic brachytherapy (TPPB) in intermediate-risk prostate cancer (PCa) patients remains controversial. Data on the efficacy of combining androgen-deprivation therapy (ADT) with ^125^I-TPPB in this group remain limited and consequently the guidelines of the American Brachytherapy Society (ABS) provide no firm recommendations.

**Methods/Design:**

Seed and Hormone for Intermediate-risk Prostate Cancer (SHIP) 0804 is a phase III, multicenter, randomized, controlled study that will investigate the impact of adjuvant ADT following neoadjuvant ADT and ^125^I-TPPB. Prior to the end of March, 2011, a total of 420 patients with intermediate-risk, localized PCa will be enrolled and randomized to one of two treatment arms. These patients will be recruited from 20 institutions, all of which have broad experience of ^125^I-TPPB. Pathological slides will be centrally reviewed to confirm patient eligibility. The patients will initially undergo 3-month ADT prior to ^125^I-TPPB. Those randomly assigned to adjuvant therapy will subsequently undergo 9 months of adjuvant ADT. All participants will be assessed at baseline and at the following intervals: every 3 months for the first 24 months following ^125^I-TPPB, every 6 months during the 24- to 60-month post-^125^I-TPPB interval, annually between 60 and 84 months post-^125^I-TPPB, and on the 10th anniversary of treatment.

The primary endpoint is biochemical progression-free survival (BPFS). Secondary endpoints are overall survival (OS), clinical progression-free survival, disease-specific survival, salvage therapy non-adaptive interval, acceptability (assessed using the international prostate symptom score [IPSS]), quality of life (QOL) evaluation, and adverse events. In the correlative study (SHIP36B), we also evaluate biopsy results at 36 months following treatment to examine the relationship between the results and the eventual recurrence after completion of radiotherapy.

**Discussion:**

These two multicenter trials (SHIP0804 & SHIP36B) are expected to provide crucial data regarding the efficacy, acceptability and safety of adjuvant ADT. SHIP36B will also provide important information about the prognostic implications of PSA levels in intermediate-risk PCa patients treated with ^125^I-TPPB.

**Trial registration:**

NCT00664456, NCT00898326, JUSMH-BRI-GU05-01, JUSMH-TRIGU0709

## Background

Studies show improved outcomes when androgen-deprivation therapy (ADT) is included in the therapeutic regimen for men with intermediate-risk prostate cancer [1], and available data provide stronger support for the benefits of ADT than for those of radiation dose escalation in patients with intermediate-risk disease [1]. In order to obtain further gain, five phase III clinical trials (RTOG0232, RTOG0815, NCT00890006, NCT00388804, NCT00005044) examining combined use of external beam radiation (EBRT) and ADT in intermediate-risk patients are being planned or currently in progress [2,3].

^125^I-transperineal prostatic brachytherapy (TPPB) was added to the Japanese armamentarium for treatment of localized PCa in 2003 [4]. Since then, more than 5,000 patients have undergone this procedure. However, the indication for brachytherapy in intermediate-risk patients remains controversial. TPPB alone is considered inadequate for these patients due to possible extraprostatic extension. Retrospective studies have suggested that ADT can improve outcomes in intermediate-risk PCa patients treated with TPPB [1]. There are, however, no reported prospective studies documenting the effectiveness of adjuvant ADT with TPPB for intermediate-risk patients. Such prospective clinical trials are clearly needed. In the present paper, we describe our study protocol for a phase III, multicenter, randomized, controlled study of TPPB with or without adjuvant ADT in patients with intermediate-risk PCa.

Adjuvant ADT is significantly associated with adverse events. The PROST-QA study prospectively measured patient-reported quality of life outcomes before and after prostate cancer treatment [5]. Sexual function was persistently worse among radiation patients who received ADT than among those who did not. Vitality and other outcomes related to hormonal therapy (e.g., fatigue, weight change, gynecomastia, depression, and hot flashes) were also worse in the ADT patients. Furthermore, symptoms persisted after radiotherapy for up to two years despite < 1 year of ADT [5]. Thus, patients at intermediate risk who already face the dilemma of choosing between treatment modalities have their choice further complicated by the adverse event profile of ADT and recommending this therapy may not be appropriate until we have firmer evidence of its benefit. The present trial (SHIP0804) is also expected to provide crucial data regarding acceptability (assessed using the international prostate symptom score [IPSS]) and quality of life (QOL).

For assessment of PCa treatment efficacy, biochemical failure is a rational early endpoint. Numerous investigators in the PSA era have demonstrated the importance of biochemical outcome following treatment. A definition of PSA nadir level + 2 ng/mL, which is adopted in the present study, was recently proposed as the recommended definition of biochemical failure [6]. This definition reduces misinterpretation of unstable PSA status, such as that due to the bounce phenomenon, and eliminates backdating of progression to preceding measurements.

On the other hand, it was previously reported that the correlation between biopsy results at 6-36 months after completion of radiotherapy and the eventual development of recurrence after completion of radiotherapy was highly significant retrospectively [7]. In a correlative study (SHIP36B), the relationship between PSA levels and biopsy results 36 months after treatment will be investigated. SHIP36B will also provide important information about the prognostic implications of PSA levels in intermediate-risk PCa patients treated with TPPB.

As mentioned above, the optimal protocol for ^125^I-transperineal prostatic brachytherapy (TPPB) in intermediate-risk prostate cancer (PCa) patients remains controversial. In the present paper, we describe our study protocols for a phase III, multicenter, randomized, controlled study of TPPB with or without adjuvant ADT in patients with intermediate-risk PCa (SHIP0804), and to examine the relationship between PSA levels and biopsy results 36 months after treatment (SHIP36B).

## Methods/Design

### Aim of the study

To examine the effect of adjuvant luteinizing hormone-releasing hormone-agonist (LHRHa) therapy (SHIP0804) and to examine biopsy results at 36 months after brachytherapy (SHIP36B) for intermediate-risk PCa patients who have received 3-month neoadjuvant LHRHa followed by ^125^I-TPPB.

### Study design

**Figure 1:**
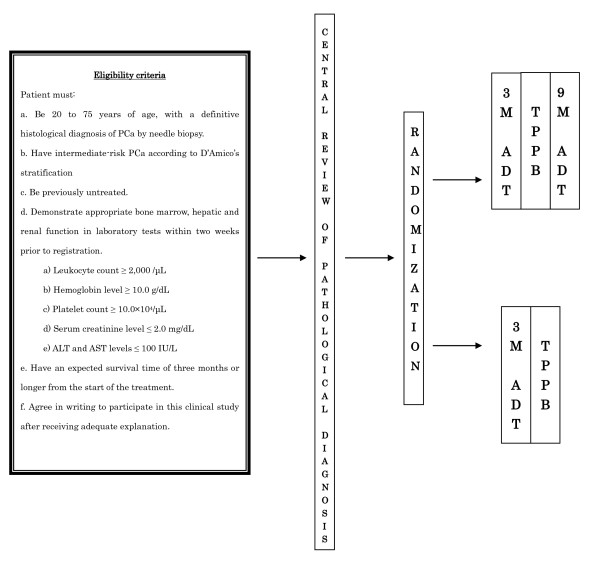
**Study design of SHIP0804 and SHIP36B. ADT, androgen deprivation therapy, TPPB, transperineal prostatic brachytherapy**.

The present investigation is a phase III, multicenter, randomized, controlled study of ADT and TPPB for patients with untreated intermediate-risk PCa (Figure 1). All patients are randomized to one of two treatment arms in which patients either do or do not receive 9-month adjuvant therapy with a LHRHa following ^125^I-TPPB. Prior to ^125^I-TPPB, all patients undergo 3-month LHRHa therapy, which consists of subcutaneous goserelin acetate, 3.6 mg/mo, or leuprolide acetate, 3.75 mg/mo. In a correlative study (SHIP36B), the relationship between PSA levels and biopsy results 36 months after treatment will be investigated.

### Additional measures

Two validated QOL questionnaires, the SF-8™, which has been translated into Japanese and is stored in the PBRN database (SQL Server 7.0), and the Expanded Prostate Cancer Index Composite (EPIC), will be administered to comprehensively cover the various aspects of physical and psychosocial well-being.

### Site selection

For an institution to participate, the institution's attending physicians must:

a. Acquire certified documentation of participation in the Japanese Brachytherapy Scientific Meeting's training session for the ^125^I-TPPB procedure.

b. Have experience with ^125^I-TPPB in at least 50 patients.

c. Regularly perform ^125^I-TPPB procedures.

### Eligibility criteria - Inclusion criteria

Patients must:

a. Be 20 to 75 years of age, with a definitive histological diagnosis of PCa by needle biopsy.

b. Have intermediate-risk PCa according to D'Amico's stratification [8]. In this stratification, patients with clinical stage T1c or T2a [9], PSA ≤ 10 ng/mL and biopsy Gleason score ≤ 6 are defined as low-risk, and patients with ≥ T2c, a PSA > 20 ng/mL or a biopsy Gleason score ≥ 8 are defined as high-risk. The remaining patients are defined as the intermediate-risk group.

c. Be previously untreated.

d. Demonstrate appropriate bone marrow, hepatic and renal function in laboratory tests within two weeks prior to registration.

a) Leukocyte count ≥ 2,000/μL

b) Hemoglobin level ≥ 10.0 g/dL

c) Platelet count ≥ 10.0 × 10^4^/μL

d) Serum creatinine level ≥ 2.0 mg/dL

e) ALT and AST levels ≥ 100 IU/L

e. Have an expected survival time of three months or longer from the start of the treatment.

f. Agree in writing to participate in this clinical study after receiving adequate explanation.

### Eligibility criteria - Exclusion criteria

Patients are ineligible if they:

a. Have previously received surgery for PCa.

b. Have PSA > 20 ng/mL.

c. Have a biopsy Gleason score ≥ 8.

e. Exhibit clinical stage ≥ T2c.

f. Have a second cancer that requires treatment.

g. Have poorly-controlled hypertension (diastolic pressure ≥ 120 mmHg)

h. Have a severe psychiatric disorder, including schizophrenia and dementia.

i. Have poorly-controlled diabetes.

j. Are using steroid drugs other than topical ointments.

k. Are using antiandrogenic therapy.

l. Are for any other reason considered by a Principal Investigator or Clinical Investigator to be inappropriate for participation in the present study.

### Informed Consent - Ethics Approval

The study was conducted in accordance with the Helsinki Declaration of 1975, as revised in 2000. All treatments for PCa are undertaken following written informed consent, and further consent is obtained for procedures to confirm the diagnosis of intermediate-risk category. This study received approval from the Foundation for Biomedical Research and Innovation, Translational Research Informatics Center (TRI) ethical review committee (approval No. 07-05, date Feb 8, 2008) and the institutional ethics committees of the participating facilities.

### Methods of recruitment and random allocation

Recruitment of patients is supported by the Seed and Hormone for Intermediate-risk Prostate Cancer Study Group (SHIP). Recruiting began in April, 2008, and is planned for completion by March, 2011. Initial recruitment is through the secretariat division of SHIP. All prostatic biopsy histological slides of newly diagnosed PCa since the study was initiated have been reviewed by central pathologists, with patient eligibility being determined at the time of review. Eligible patients are randomly assigned to one of two treatment arms through the data center at the TRI. Randomization is done centrally using a minimization method to obtain good between-group balance for factors including age category (< 70/≥ 70), PSA category (< 10/≥ 10 ng/mL) and institutions.

### Central review of the pathological diagnosis

Following enrolment of the patients, biopsy specimens are collected and forwarded to the central pathology group for review. Three uropathologists review the pathology specimens and classify each according to the modified Gleason grading system proposed by the International Society of Urological Pathology (ISUP) [10]. All cases are reviewed independently without clinical information and decisions are made by consensus.

### Technique of ^125^I-TPPB

^125^I-TPPB for all patients is administered using an ultrasound-guided technique with the Mick applicator [4]. The implant is planned to deliver a dose of at least 144Gy to the clinical target volume, which includes the prostate gland and treatment margin [11]. Although individual technical aspects are institution-dependent, efforts are made to assure optimal quality control of the radiation dose. Computed tomography images, taken at 2-5 mm intervals, are obtained one month after ^125^I-TPPB to determine the extent of edema. Dose-volume histograms for the prostate, urethra, and rectum are computed to obtain post-planning distribution data. V100 and D90 should be over 95% and 144Gy respectively for the clinically targeted volume [11,12]. We organized a quality control committee for this study with the aim of assessing inter-institutional variance of post-implant dosimetry. This board will meet regularly while this protocol is running to monitor and compare dosimetry. Results of the comparative analysis will be reported separately.

### Data collection

This design was chosen to ensure accurate, standardized and high-quality data collection. All patients giving written informed consent to the study are asked to complete a short family history and epidemiology questionnaire. Electronic Data Capture (EDC) systems are used to collect clinical data in electronic format, with clinical data being obtained from patient medical records by the TRI. A follow-up data form is completed by the Clinical Trials Practitioner (CTP) at diagnosis, 3 months, 6 months, 9 months, 12 months, 15 months, 18 months, 21 months, 24 months, and then every 6 months until 60 months post-treatment, after which forms are completed annually until 84 months from the date of ^125^I seed implantation. These forms capture information regarding patient characteristics, disease presentation, diagnosis and treatment, PSA, recurrence and survival. Annual follow up is continued until death, loss to follow up or the end of the active phase of the study (March, 2021).

### Definition of endpoints

The primary endpoint is biochemical progression-free survival (BPFS). Biochemical progression is defined as an increase in prostate specific antigen (PSA) of 2 ng/mL or greater from the nadir value following treatment. Secondary endpoints include OS, clinical progression-free survival (local, distant failure), disease-specific survival, salvage therapy non-adaptive interval, acceptability (assessed by the international prostate symptom score [IPSS]), quality of life (QOL) and adverse events. Overall survival and progression-free survival are respectively calculated from the 1st day of treatment to any death, or to identification of disease progression or death. Local progression is defined as reappearance of local tumorat the primary site. Reappearance of local tumor will be confirmed by rectal examination and imaging studies such as magnetic resonance imaging or computed tomography. The primary endpoint of a correlative study (SHIP36B) is the biopsy results at 36 months following treatment to examine the relationship between results and eventual recurrence after completion of radiotherapy.

### Planned statistical analyses

It has been shown that the 5-year BPFS rate ranges from 63% to 98% in patients with intermediate-risk PCa who undergo radiotherapy [13,14]. Assuming that the 7-year BPFS rate of the control group is 60% (the 5-year BPFS is about 69%) and the expected 7-year BPFS rate of the adjuvant ADT group is 73.6% (i.e. hazard ratio is 0.6), 190 patients for each group are needed to detect a significant difference between treatments by log-rank test with a significance level of 0.05 and a power of 80%. Given the further assumption that approximately 10% of randomized patients will be unevaluable for various reasons, the target sample size was set at 210 patients per group (420 patients total).

Statistical analyses will be performed on an intention-to-treat basis. Survival curves will be estimated using the Kaplan-Meier method. To test for differences in survival curves between the two groups of patients, the log-rank test will be used. The hazard ratio will be estimated using the Cox proportional hazard model. The longitudinal change of QOL scores (IPSS, SF-8, and EPIC) between diagnosis and 60 months following ^125^I-TPPB will also be compared between groups. Patients will be evaluated for toxicity, graded according to the National Cancer Institute Common Toxicity Criteria version 3.0 [15]. For all patients, the incident proportion of grade 3 adverse events will be compared between groups by Fisher exact test. All tests will be two-sided, and a value of 0.05 will be considered statistically significant. Five years after the last patient is recruited, an interim analysis will be performed and the results will be reported to the Independent Data Monitoring Committee.

### Patient enrollment and anticipated completion of enrollment

Monthly enrollment was moderately below goal, but cumulative enrollment reached 181 cases in December, 2009. Slow enrollment may reflect the relatively low frequency of PCa patients with intermediate-risk [4]. Our current expectation is that the final patient will be enrolled by March, 2011; the study will be clinically complete by 2021 and results will be available during the first quarter of 2022.

## Discussion

There are considerable data supporting the use of adjuvant ADT with EBRT in selected patients with PCa, particularly those with locally advanced, unfavorable-risk disease [1,16,17]. Presently, however, the effect of combination therapy with ADT in localized prostate cancer remains controversial. A prospective randomized controlled trial including 206 intermediate- to high-risk, localized PCa patients (PSA ≥ 10 ng/mL, Gleason score ≥ 7, or radiographic evidence of extraprostatic disease) indicated 6-month ADT (neoadjuvant, concurrent and adjuvant) in combination with EBRT led to significantly higher overall survival than RT alone [17]. However, the study enrolled a mixed population of patients at varying risks of disease recurrence, complicating extrapolation of the results to any single risk group.

The ABS recommends neoadjuvant ADT in conjunction with TPPB for downsizing the prostatic gland when the initial size surpasses 60 cc, but provides no clear indication for using ADT adjuvantly in intermediate- to high-risk disease. The liberal use of neoadjuvant therapy can be a confounder when evaluating efficacy of ADT in combination with TPPB within a certain risk category. The uniformly used neoadjuvant ADT and the consequent downsizing of the gland will facilitate recruitment and reduce potential bias in patient selection in our randomized trial. Since the participating institutions were overloaded with a long waiting list of patients for TPPB, it was considered more practical to have certain period of neoadjuvant ADT prior to TPPB. All patients are thus planned to undergo fixed-term, 3-month neoadjuvant ADT.

Some retrospective studies suggest beneficial effects for combined use of ADT with TPPB and/or EBRT in PCa patients with unfavorable features [18,19]. However, there are no reports of prospective studies documenting the effectiveness of adjuvant ADT with TPPB for intermediate-risk PCa patients. The study by D'Amico et al showed the benefits of a relatively short, 6-month course of ADT [17]. We accordingly set the adjuvant ADT administration period at 9 months to facilitate detection of any differences between groups receiving or not receiving this therapy. Adverse effects will be assessed using validated questionnaires.

In intermediate-risk PCa patients, according to Partin's table [20], the probabilities for pT3, extracapsular extension (ECE), seminal vesicle involvement (SVI) and lymph node (LN) involvement are predicted to be 58 to 82%, 40 to 57%, 11 to 23% and 6 to 29%, respectively. Naito's Japanese nomogram predicts the respective corresponding rates as 39 to 76%, 33 to 59%, 5 to 10% and 1 to 7% [21]. This patient population therefore harbors a definite risk of pathologically more advanced disease including the possibility of subclinical metastatic disease. Radiation dose escalation presumes that higher radiation doses administered to the prostate and periprostatic area are more likely to eradicate prostate cancer cells than lower doses of radiation [22]. However, the high intraprostatic dose coverage provided by ^125^I-TPPB may be insufficient for periprostatic disease [19,23]. Our study design will provide additional insight regarding the efficacy and limitations of TPPB when adjuvant ADT is applied.

The primary goal of SHIP0804 is to evaluate the effectiveness of adjuvant ADT following TPPB on the 10-year BPFS endpoint in intermediate-risk PCa. Data from this clinical trial may also confirm PSA as an appropriate surrogate endpoint after treatment of PCa.

It was reported that the postirradiation biopsy performed at a sufficient interval after radiotherapy can provide accurate prognostic information useful in the determination of the success or failure of radiotherapy in an individual patient as well as the measurement of overall efficacy of any particular radiotherapeutic regimen [7]. In a retrospective analysis, Scardino et al. [7] previously reported that by analyzing the 140 patients who had one or more needle biopsies performed 6-36 months after completion of radiotherapy, 32% patients had one or more biopsies positive for cancer, and concluded that the correlation between biopsy results and the eventual development of recurrence was highly significant. Stone et al. [24] also reported that by analyzing biopsy results in 508 patients with prostate cancer treated with brachytherapy, cancer positive biopsy was associated with high PSA, stage, risk, and no and low dose hormonal therapy. Recently, the results of prostate biopsies 24 months following radiation were also found to be strongly predictive of subsequent disease-free survival in a Canadian randomized trial [25]. In our correlative study (SHIP36B), histological effects of ^125^I-TPPB and ADT in relation to PSA levels and kinetics will be investigated separately. This exploratory design was determined since the organizing committee could not reach the consensus to investigate its impact under randomized scheme. Nevertheless, SHIP36B will provide important further information about the prognostic implication of PSA levels in intermediate-risk PCa patients treated with ^125^I-TPPB.

## Abbreviations

ABS: American Brachytherapy Society; BPFS: biochemical progression-free survival; CTP: Clinical Trials Practitioner; IPSS: International Prostate Symptom Score; EBRT: external beam radiation therapy; EPIC: Expanded Prostate Cancer Index Composite; OS: overall survival; LHRHa: luteinizing hormone-releasing hormone agonist; PCa: prostate cancer; PSA: prostate specific antigen; TRI: Translational Research Informatics Center; TRUS: transrectal ultrasound; QOL: quality of life.

## Competing interests

The authors declare that they have no competing interests.

## Authors' contributions

KMik, TD, HY, MF and SE planned, coordinated and conducted the study. Medical care was provided by KMik, HS, MK, MA and SE. KMiy provided randomization. HT contributed pathological diagnosis. TK and MF took part in conducting the study. The scientific program was planned by KMik, MF and SE, and carried out by KMik. All authors read and approved the final manuscript. All other participants in this study contribute to the enrollment, treatment and follow up of patients.

## Appendix

The following individuals and institutions participated in this study of seed and hormone for intermediate-risk prostate cancer (SHIP0804 and SHIP36B): K. Miki, MD, H. Sasaki, MD, M. Kido, MD, S. Egawa, MD, Department of Urology, Jikei University School of Medicine (JU), H. Takahashi, MD, Department of Pathology, Jikei University School of Medicine (JP) and M. Aoki, MD, Department of Radiology, Jikei University School of Medicine (JR), Tokyo, Japan; T. Kiba, MD, K. Miyakoda, MPH, M. Fukushima, MD, Translational Research Informatics Center (TRI), Kobe, Japan; H. Yamanaka, MD, Institutes of Preventive Medicine, Kurosawa Hospital (KH), Takasaki, Japan; T. Dokiya, MD, Department of Radiation Oncology, Saitama Medical University International Medical Center (SR), Hidaka, Japan; Y. Nasu, MD, Okayama University Graduate School of Medicine, Okayama, Japan; T. Yamashita, MD, Cancer Institute Hospital, Tokyo, Japan; K. Ito, MD, Gunma University Graduate School of Medicine, Maebashi, Japan; T. Satoh, MD, Kitasato University School of Medicine, Sagamihara, Japan; H. Kanayama, MD, University of Tokushima, Tokushima, Japan; T. Fukagai, MD, Showa University School of Medicine, Tokyo, Japan; S. Naito, MD, Kyushu University, Fukuoka, Japan; T. Miki, MD, Kyoto Prefectural University of Medicine, Kyoto, Japan; H. Sakai, MD, Nagasaki University School of Medicine, Nagasaki, Japan; Y. Kakehi, MD, Kagawa University, Kida, Japan; T. Akimoto, MD, Tokyo Women's Medical University, Tokyo, Japan; S. Saito, MD, National Hospital Organization Tokyo Medical Center, Tokyo, Japan; M. Hashine, MD, Shikoku Cancer Center, Matsuyama, Japan; N. Tanaka, MD, Nara Medical University, Kashihara, Japan; H. Uemura, MD, Yokohama City University, Yokohama, Japan; M. Nakano, MD, Gifu University, Gifu, Japan; T. Monma, MD, National Hospital Organization Saitama National Hospital, Wako, Japan; G. Kimura, MD, Nippon Medical School, Tokyo, Japan.

## Pre-publication history

The pre-publication history for this paper can be accessed here:

http://www.biomedcentral.com/1471-2407/10/572/prepub
